# Effect of Glazing on Flexural Strength of Full-Contour Zirconia

**DOI:** 10.1155/2018/8793481

**Published:** 2018-02-05

**Authors:** Hattanas Kumchai, Patrapan Juntavee, Arthur F. Sun, Dan Nathanson

**Affiliations:** Department of Restorative Sciences & Biomaterials, Boston University Henry M. Goldman School of Dental Medicine, Boston, MA, USA

## Abstract

**Objective:**

The purpose of this study was to evaluate the effect of glazing on flexural strength of highly translucent zirconia materials.

**Materials and Methods:**

Specimens of three brands of zirconia bars (Prettau Zirconia, Zirkonzahn; inCoris TZI, Sirona; and Zirlux FC, Pentron Ceramics) were prepared and polished according to manufacturers' instructions. Final specimen dimensions were 20 × 4 × 2 mm. The specimens from each brand were divided into 3 groups (*N* = 10): control, heat-treated, and glazed. Heat-treated specimens were fired without the application of the glaze material. The glaze material was applied to the glazed specimens before being fired. A three-point bending test (15 mm span) was performed in an Instron universal testing machine (ISO 6872). Data were analyzed by ANOVA and Tukey's HSD post hoc test (*α* = 0.05).

**Results:**

Two-way ANOVA showed a significant influence of surface treatments on flexural strength of zirconia materials (*P* ≤ 0.05). There was no significant difference in flexural strength among the different brands of highly translucent zirconia (*P* ≥ 0.05). Tukey's HSD post hoc test showed that specimens in the “glazed” group had significantly lower flexural strength than the control and heat-treated groups (*P* ≤ 0.05).

**Conclusion:**

Within the limitations of the study, external glazing decreased the flexural strength of highly translucent zirconia.

## 1. Introduction

The glazing of porcelain dental restorations is a routine procedure designed to provide esthetic and hygienic glass-coated surfaces on the finished restoration [1, 2]. Applied overglaze is a low-fusing clear porcelain that is painted onto the surface of the restoration and fired at a fusing temperature much lower than that of the dentin and enamel porcelain [3]. Glazing for the purpose of strengthening brittle ceramics can be considered the production of a surface layer of lower thermal expansion glass that serves many functions when cooled. It places the surface in a compressive state. The thin layer of glass also reduces the depth and width of surface flaws and could theoretically strengthen the material [4]. Glazed surfaces result in less plaque accumulation. In addition, glazed porcelain can imitate the gloss and characterization of the natural tooth [5]. It decreases the exposure of the dental restoration to the oral cavity and provides the necessary smoothness [2].

However, many studies have shown that glazing does not increase flexural strength [1, 4, 6]. It has also been shown that autoglazing does not cause a difference in the flexural strength of porcelain specimens [1, 4, 6]. Although glazing reduces the wear of opposing enamel, it was found that glazing causes cracks in the porcelain and thus decreases flexural strength [4]. Yener et al. [7] compared the biaxial flexural strength of three different brands of zirconia (Zirkonzahn, Cercon, and Ceramill) with and without glazing. Their results showed that glazing significantly decreased the flexural strength for all systems.

The introduction of highly translucent yttrium oxide-stabilized tetragonal zirconia polycrystals (Y-TZPs) enabled the esthetics of monolithic zirconia restorations to be sufficiently improved. The high translucency of the Y-TZP was achievable by decreased alumina content [8], which is normally incorporated into zirconia materials to increase their stability during aging yet causes light scattering due to a refractive index different from that of zirconia. Reduction or elimination of alumina content in zirconia materials makes them vulnerable to problems with stability during aging [9].

Although many clinicians prefer to glaze monolithic zirconia restorations, data regarding the effect of glazing on the mechanical properties of these materials are still lacking. The purpose of this study was to evaluate the effect of glazing on the flexural strength of highly translucent zirconia materials. The study examined the flexural strength of three different brands of translucent zirconia with different surface treatments.

## 2. Materials and Methods

Three different brands of zirconia and two different glaze materials (Table 1) were tested in this study. In total, 10 rectangular specimens of zirconia for each group were prepared (dimensions, 2.5 × 5 × 25 mm) by means of a 15LC diamond-wafering blade mounted on an Isomet 2000 precision saw (Buehler, Lake Bluff, Illinois, USA). The cuts were made at 800 rpm with a 300-gram load, with cooling provided by a dual-nozzle water irrigation system. Specimen dimensions were verified after they were sectioned by means of a Mitutoyo absolute IP-67 digital vernier caliper (Mitutoyo, Kawasaki, Japan).

Each specimen was polished and beveled (45°, 0.15 mm edge chamfer) in a Buehler Ecomet 250 grinder/polisher (Buehler, Lake Bluff, Illinois, USA). The polishing was then done with a 15-micron grit diamond polishing pad at 30 rpm with water irrigation for 90 s for each side, after which the specimen was rinsed thoroughly. For beveling, the specimen was placed at 45° on a 15-micron grit diamond polishing pad at 30 rpm with water irrigation. The specimen was inspected for the presence of chamfer edge after the beveling. All specimens were air-dried at room temperature for at least 24 h to minimize the possibility of water being trapped in the zirconia structure. The sectioned bars were sintered with the parameters as in Table 2.

After being sintered, the specimens were polished again in a Buehler Ecomet 250 grinder/polisher (Buehler, Lake Bluff, Illinois, USA). The polishing was accomplished with a 15-micron grit diamond polishing pad, followed by a 6 *μ*m polycrystalline diamond suspension with a polishing pad, respectively, at 30 rpm with water irrigation (90 s for each side), and then thoroughly rinsed. The dimensions of the bars after being sintered and polished were approximately 20 × 4 × 2 mm according to ISO 6872:2015.

Specimens were divided into different groups according to surface treatments: (a) no treatment, (b) heat-treated, and (c) glazed.

### 2.1. Heat-Treated Firing

Zirconia specimens were air-dried and placed on a firing tray. They were then fired with the glaze firing cycle but without glaze materials. The firing parameters are shown in Table 3.

### 2.2. Glazing

Zirconia specimens were air-dried. The overglaze pastes were mixed with glaze liquids and applied in a thin coat on the entire surface of each zirconia bar using a ceramic brush. For Prettau Zirconia and inCoris TZI specimens, Zirkonzahn glaze paste and Zirkonzahn ICE stain liquid were used. For Zirlux FC specimens, Zirlux FC glaze paste and universal liquid were used. The glazing group specimens were then placed on a firing tray. Glaze firing was done according to manufacturers' instructions (Table 4).

All specimens were then stored in 37°C deionized water for 24 h before testing began. Specimens were tested for flexural strength on a three-point bending test conducted on an Instron 5566A universal testing machine (Instron, Norwood, Massachusetts, USA) with a 1 kN load cell at a crosshead speed of 0.5 mm/min. Each specimen was loaded to failure and failure load data was obtained. The data were then calculated into flexural strength according to the following formula:

(1) $$\begin{array}{c} \sigma =\frac{3FL}{2bd^{2}}, \end{array}$$

where *F* is the failure load (force) at the fracture point (*N*), *L* is the length of the support span, *b* is the width, and *d* is the thickness.

Statistical analysis was performed by means of SPSS System 23 for Windows. The means of each group were analyzed by two-way ANOVA, with flexural strength as the dependent variable and the zirconia systems and surface treatments as the independent factors. *P* values less than 0.05 were considered statistically significant in all tests. Data from all zirconia brands were pooled, and multiple comparisons between and among different surface treatments were evaluated by Tukey's HSD test.

## 3. Results

**Figure 1:**
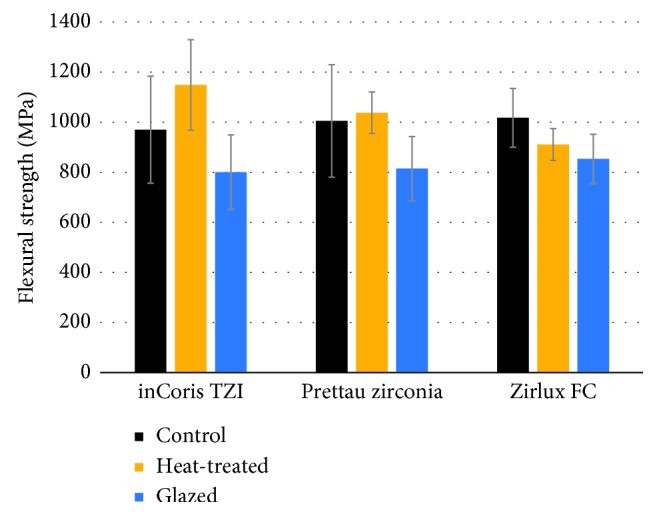
Flexural strength of highly translucent zirconia with different surface treatments.

**Figure 2:**
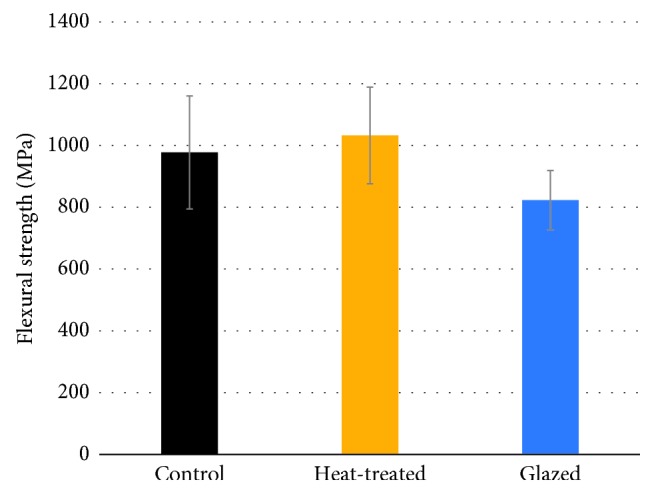
Pooled data from mean flexural strengths of highly translucent zirconia materials with different surface treatments.

The flexural strength data obtained from the three-point bending tests are presented in Table 5, Figures 1 and 2. The results of two-way ANOVA showed the significant influence of surface treatments on flexural strength (*P* < 0.05). The Tukey HSD post hoc test showed that specimens in the glazed group had significantly lower flexural strength than did those in the control and heat-treated groups (*P* < 0.05). There was no significant difference in flexural strength between specimens in the control and heat-treated groups (*P* > 0.05). There was no significant influence of zirconia brands on flexural strength (*P* > 0.05).

## 4. Discussion

Glazing after grinding is believed to increase the strength of a ceramic restoration because it decreases the depths of surface cracks [4]. However, the strengthening effect of glazing on porcelain is not clearly understood [4, 10].

In this study, for investigation of the effect of glaze on the flexural strength of three different highly translucent zirconia systems, Prettau Zirconia, inCoris TZI, and Zirlux FC were used. Glazes were applied according to the manufacturer's recommendations for each system. It has been reported that 0.05 mm glaze thickness is sufficient to prolong its integrity [11]. Therefore, a 0.05 mm thickness of glaze was applied to each surface (a total of 0.1 mm) of bar-shaped specimens.

Statistical analysis showed that glazing decreased the flexural strength of the highly translucent zirconia. This finding was supported by the results from another study [7], reporting that glazing decreased the biaxial flexural strength of Zirkonzahn, Cercon, and Ceramill zirconia.

We conducted this study on heat-treated groups to determine whether the glaze-firing cycle had an effect on the flexural strength of zirconia. We found that there was no significant reduction in the flexural strength of zirconia after being fired with a glazing cycle without glazing materials. The only difference between specimens in this group and those in the glazed group was the absence of glaze materials (a mixture of glaze powder and liquid). Therefore, the strength reduction in glazed zirconia found in this study might be due to the glazing.

Residual stresses played an important role in determining the strength of ceramic materials. In cases of residual tensile stress, preexisting stress will amplify the applied cycling stress and induce cracks in that region. Compressive global residual stresses within the ceramic surface can somehow strengthen the material; however, excessive compressive residual stresses can cause lateral cracks to grow and propagate on the surface, and these will eventually cause the material to fail. According to Swain [12], residual stresses can be introduced during the firing process due to thermal expansion mismatch and tempering stresses associated with temperature gradients during cooling.

Due to the metastability of tetragonal zirconia, stress-generating surface treatments such as grinding or sandblasting are also capable of triggering the *t*→*m* transformation with the associated volume increase [13]. In this study, we polished all zirconia bars before performing any surface treatment. Therefore, we expected that there would be some *t*→*m* transformation during this procedure. The coefficient of thermal expansion (CTE) of tetragonal zirconia is approximately 10.5 × 10^−6^·K^−1^, while the CTE of monoclinic zirconia is only 7.5 × 10^−6^·K^−1^. Therefore, the CTE of polished zirconia might depend on the degree of phase transformation brought about by polishing. The CTE of porcelain was also believed to be changed during firing. Since glazed materials consist of porcelain powder (∼60%), the CTE change in porcelain might occur in glazed materials during porcelain firing.

Tempering stresses associated with temperature gradients during cooling have also been reported to cause residual stress on ceramics. The poor thermal conductivity of porcelain and zirconia, which is much lower than that of metal alloys, combined with poor thermal diffusivity [14], results in a high temperature difference through the specimens, resulting in high residual tempering stresses and thermal gradients.

The composition of the highly translucent zirconia materials used in this study was different from that of the conventional zirconia designed for coping or a fixed dental prosthesis (FDP) framework. The most notable difference is the reduction or absence of alumina, which is reported to play a significant role in phase stabilization. The effect of residual stresses on the mechanical properties of highly translucent zirconia can be more than that of conventional zirconia. Crown geometry, as is commonly understood, affects residual stress. The scenario of crowns may never be captured in its entirety with the use of simple bar models as a result of their multifaceted geometric properties.

It would be particularly useful in understanding the stress mechanic of this study. Fractographic analysis can give useful information regarding failure origin, failure stress, fracture toughness, and residual stress. Further investigations including fractographic analysis and effect of crown geometry are recommended to complement the present study.

## 5. Conclusions

Within the limitations of the study, the following conclusions can be drawn:

1. Glazing decreased the flexural strength of the highly translucent zirconia tested (*P* < 0.05).
2. Glaze firing without the glaze material had no effect on the flexural strength of the highly translucent zirconia tested (*P* < 0.05).
3. There was no significant difference in the flexural strengths of different brands of the highly translucent zirconia tested (*P* > 0.05).

## Figures and Tables

**Table 1:** Materials used in this study.

Material	Brand name
Zirconia	inCoris TZI (Sirona, Germany)
	Prettau Zirconia (Zirkonzahn, Germany)
	Zirlux FC (Pentron Ceramics, California)
Glaze	Zirkonzahn glaze paste + liquid (Zirkonzahn, Germany)
	Zirlux FC glaze paste + liquid (Pentron Ceramics, California)

**Table 2:** Firing parameters for zirconia sintering.

Material	Entry temperature (°C)	Rising temperature rate (°C)	Final temperature (°C)	Holding time (min)
inCoris TZI	Room temperature	25	1530	120
Prettau Zirconia	Room temperature	10	1600	120
Zirlux FC	Room temperature	10	1500	120

**Table 3:** Firing steps for heat-treated firing.

Brand	Idle temp. (°C)	Dry time (s)	End temp. (°C)	Holding time (min)	Heat rate (°C/min)	Vacuum	Tray open (°C)
Prettau Zirconia	350	5	820	2	55	+	—
inCoris TZI	350	5	820	2	55	+	—
Zirlux FC	—	360	1000	0	55	+	480

**Table 4:** Firing steps for glazed firing.

Glaze material	Idle temp. (°C)	Dry time (s)	End temp. (°C)	Holding time (min)	Heat rate (°C/min)	Vacuum	Tray open (°C)
Prettau Zirconia glaze	350	5	820	2	55	+	—
Zirlux FC glaze	—	6	1000	0	55	+	480

**Table 5:** Flexural strength of highly translucent zirconia with different surface treatments.

Zirconia	Flexural strength (MPa) ± standard deviation		
	Control	Heat-treated	Glazed
inCoris TZI	970.17 ± 213.55	1148.86 ± 224.45	800.88 ± 117.12
Prettau Zirconia	1005.12 ± 180.85	1037.80 ± 82.81	815.00 ± 63.78
Zirlux FC	1017.36 ± 148.69	911.03 ± 127.67	853.62 ± 98.55
